# Poly[[diaqua­(μ_8_-benzene-1,2,4,5-tetra­carboxyl­ato)calciumzinc] monohydrate]

**DOI:** 10.1107/S1600536812034113

**Published:** 2012-08-04

**Authors:** Yong-Yan Jia, Bo Chen, Yu-Xia Yuan

**Affiliations:** a Pharmacy College, Henan University of Traditional Chinese Medicine, Zhengzhou 450008, People’s Republic of China

## Abstract

In the title complex, {[CaZn(C_10_H_2_O_8_)(H_2_O)_2_]·H_2_O}_*n*_, the Zn^II^ ion is coordinated by four O atoms from four benzene-1,2,4,5-tetra­carboxyl­ate anions in a distorted tetra­hedral geometry. The Ca^II^ ion is eight-coordinated by six O atoms from four benzene-1,2,4,5-tetra­carboxyl­ate anions and by two water mol­ecules in a distorted square-anti­prismatic geometry. The Ca^II^ and Zn^II^ ions and the lattice water mol­ecule are located on twofold rotation axes; the centroid of the benzene-1,2,4,5-tetra­carboxyl­ate anion is located on a centre of inversion. The μ_8_-bridging mode of the anion results in the formation of a three-dimensional structure with channels extending along [100] in which lattice water mol­ecules are situated. Inter­molecular O—H⋯O hydrogen bonds involving the coordinating and lattice water mol­ecules as donors and the carboxyl­ate O atoms and lattice water mol­ecules as acceptors are present in the structure.

## Related literature

For background to complexes based on benzene-1,2,4,5-tetra­carb­oxy­lic acid and its anions, see: Prajapati *et al.* (2009 ▶); Xie *et al.* (2008 ▶).
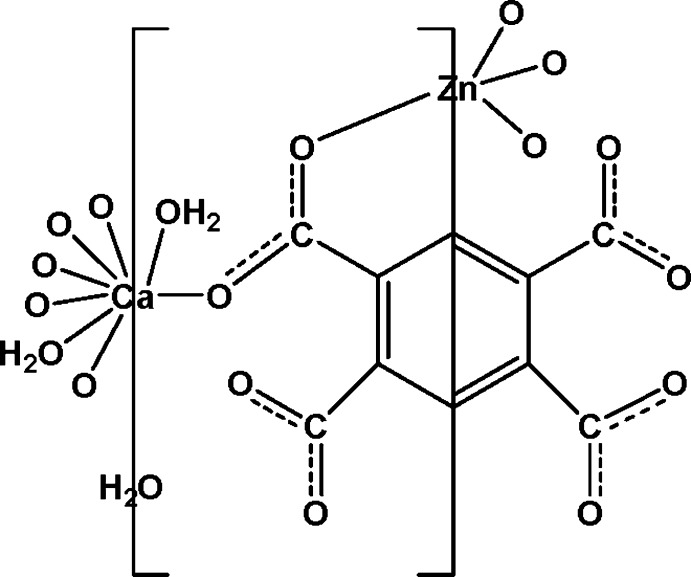


## Experimental

### 

#### Crystal data

[CaZn(C_10_H_2_O_8_)(H_2_O)_2_]·H_2_O
*M* *_r_* = 409.61
Monoclinic, 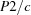

*a* = 6.2006 (12) Å
*b* = 9.770 (2) Å
*c* = 11.259 (3) Å
β = 115.33 (2)°
*V* = 616.5 (2) Å^3^
*Z* = 2
Mo *K*α radiation
μ = 2.47 mm^−1^
*T* = 293 K
0.19 × 0.17 × 0.14 mm

#### Data collection

Rigaku Saturn CCD diffractometer
Absorption correction: multi-scan (*CrystalClear*; Rigaku/MSC, 2004 ▶) *T*_min_ = 0.651, *T*_max_ = 0.723
3849 measured reflections
1447 independent reflections
1360 reflections with *I* > 2σ(*I*)
*R*_int_ = 0.027

#### Refinement

*R*[*F* ^2^ > 2σ(*F* ^2^)] = 0.027
*wR*(*F* ^2^) = 0.099
*S* = 0.84
1447 reflections
106 parameters
H-atom parameters constrained
Δρ_max_ = 0.38 e Å^−3^
Δρ_min_ = −0.56 e Å^−3^

### 

Data collection: *CrystalClear* (Rigaku/MSC, 2004 ▶); cell refinement: *CrystalClear*; data reduction: *CrystalClear*; program(s) used to solve structure: *SHELXS97* (Sheldrick, 2008 ▶); program(s) used to refine structure: *SHELXL97* (Sheldrick, 2008 ▶); molecular graphics: *SHELXTL* (Sheldrick, 2008 ▶); software used to prepare material for publication: *publCIF* (Westrip, 2010 ▶).

## Supplementary Material

Crystal structure: contains datablock(s) global, I. DOI: 10.1107/S1600536812034113/wm2661sup1.cif

Structure factors: contains datablock(s) I. DOI: 10.1107/S1600536812034113/wm2661Isup2.hkl

Additional supplementary materials:  crystallographic information; 3D view; checkCIF report

## Figures and Tables

**Table 1:** Hydrogen-bond geometry (Å, °)

*D*—H⋯*A*	*D*—H	H⋯*A*	*D*⋯*A*	*D*—H⋯*A*
O6—H6⋯O4	0.85	2.49	3.255 (4)	151
O5—H5*A*⋯O6^i^	0.85	2.11	2.914 (4)	157
O5—H5*B*⋯O1^ii^	0.85	2.16	2.951 (2)	156

Symmetry codes: (i) 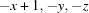; (ii) 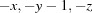.

**Fig. 1.**
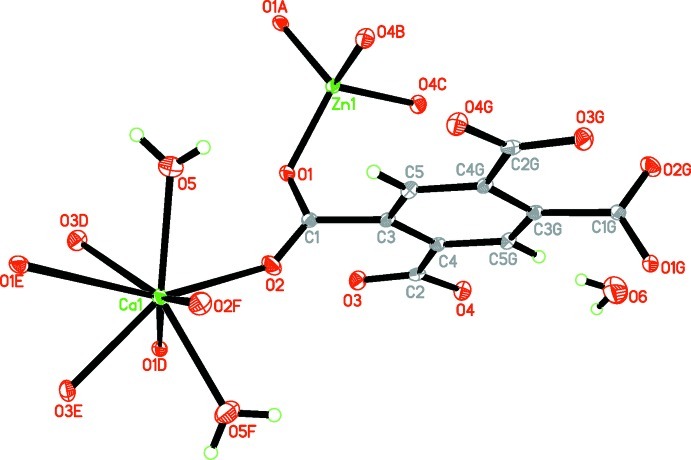
View of the building units of the title compound showing the coordination environment around the metal ions. The displacement parameters of the atoms are displayed at the 30% probability level. [Symmetry code A: -x, y, -z - 1/2; B: x - 1, y, z; C: -x + 1, y, -z - 1/2; D: -x + 1, -y, -z - 1; E: x, -y - 1, z + 1/2; F: -x + 1, y, 1/2 - z; G: -x + 1, -y, -z.].

**Fig. 2.**
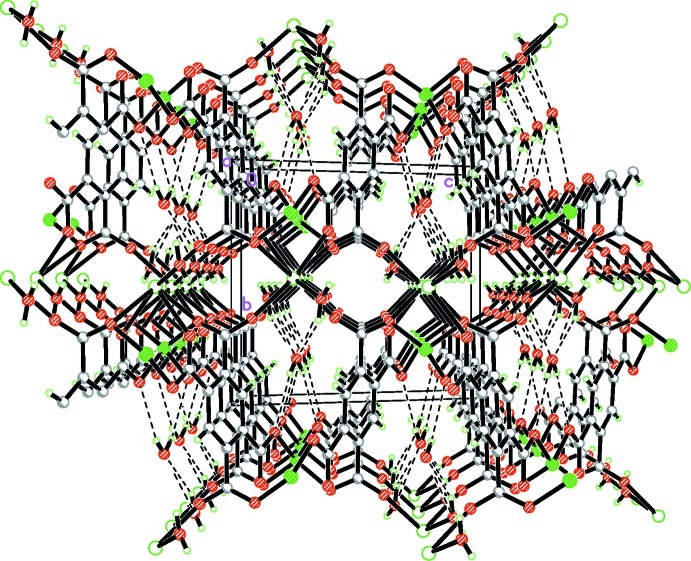
Packing plot of the title complex with O—H···O hydrogen bonds indicated by dashed lines.

## supplementary crystallographic information

### Comment

A large number of complexes constructed from the multidentate aromatic ligand benzene-1,2,4,5-tetracarboxylic acid and its corresponding anions have been extensively studied due to the diversity of the coordination modes and sensitivity to pH values of the carboxylate anions. Some of the final products exhibit useful functional properties (Prajapati *et al.*, 2009; Xie *et al.*, 2008). In order to further explore complexes with new structures, we selected benzene-1,2,4,5-tetracarboxylic acid as precursor to self-assembly with ZnCl_2_ and CaCl_2_ simultaneously in solution and obtained the title complex, [CaZn(C_10_H_2_O_8_)(H_2_O)_2_]^.^(H_2_O), the crystal structure of which is reported herein.

As shown in Figure 1, the Zn^II^ ion displays a distorted tetrahedral coordination geometry defined by four oxygen atoms from four symmetry-related benzene-1,2,4,5-tetracarboxylate groups (O1, O1A, O4B and O4C). The Ca^II^ ion is bound to six oxygen atoms from four benzene-1,2,4,5-tetracarboxylate groups (O1E, O1D, O2, O2F, O3D, O3E) and two water molecules (O5, O5F) leading to a distorted square-antiprismatic geometry. The base plane of the square antiprism consists of atoms O1E, O2F, O3E, and O5F with a mean deviation of 0.1065 Å from the least-squares plane. The top plane of the square antiprism consists of symmetry-related atoms O1D, O2, O3D, and O5. The dihedral angle between the two planes is 3.9 °. The three-dimensional set-up of the structure leaves space for channels extending along [100] where the lattice water molecules are located.

Intermolecular O—H···O hydrogen bonds between coordinating water molecules and lattice water molecules, between coordinating water molecules and carboxylate groups, and between solvent water molecules and carboxylate groups consolidate the crystal packing (Table 1, Fig. 2).

### Experimental

A mixture of ZnCl_2_ (0.05 mmol), CaCl_2_ (0.05 mmol), benzene-1,2,4,5-tetracarboxylic acid (0.05 mmol), water (4 ml) and methanol (4 ml) was placed in a 25 ml Teflon-lined stainless steel vessel and heated at 393 K for 72 h, then cooled to room temperature. Colourless crystals were obtained from the filtrate and dried in air.

### Refinement

The H atom bound to C5 was positioned geometrically and refined as riding, with C—H = 0.93 Å. H atoms bound to water O atoms were found from difference maps and refined with distance restraints of O—H = 0.85 Å. All H atoms were refined with U_iso_(H) = 1.2 U_eq_(C,O).

### Crystal data

**Table:**

[CaZn(C_10_H_2_O_8_)(H_2_O)_2_]·H_2_O	*F*(000) = 412
*M**_r_* = 409.61	*D*_x_ = 2.207 Mg m^−^^3^
Monoclinic, *P*2/*c*	Mo *K*α radiation, λ = 0.71073 Å
*a* = 6.2006 (12) Å	Cell parameters from 1852 reflections
*b* = 9.770 (2) Å	θ = 2.1–27.9°
*c* = 11.259 (3) Å	µ = 2.47 mm^−^^1^
β = 115.33 (2)°	*T* = 293 K
*V* = 616.5 (2) Å^3^	Prism, colourless
*Z* = 2	0.19 × 0.17 × 0.14 mm

### Data collection

**Table:**

Rigaku Saturn CCD diffractometer	1447 independent reflections
Radiation source: fine-focus sealed tube	1360 reflections with *I* > 2σ(*I*)
Graphite monochromator	*R*_int_ = 0.027
Detector resolution: 28.5714 pixels mm^-1^	θ_max_ = 27.9°, θ_min_ = 2.1°
ω scans	*h* = −8→7
Absorption correction: multi-scan (*CrystalClear*; Rigaku/MSC, 2004)	*k* = −12→12
*T*_min_ = 0.651, *T*_max_ = 0.723	*l* = −14→9
3849 measured reflections	

### Refinement

**Table:**

Refinement on *F*^2^	Primary atom site location: structure-invariant direct methods
Least-squares matrix: full	Secondary atom site location: difference Fourier map
*R*[*F*^2^ > 2σ(*F*^2^)] = 0.027	Hydrogen site location: inferred from neighbouring sites
*wR*(*F*^2^) = 0.099	H-atom parameters constrained
*S* = 0.84	*w* = 1/[σ^2^(*F*_o_^2^) + (0.1*P*)^2^] where *P* = (*F*_o_^2^ + 2*F*_c_^2^)/3
1447 reflections	(Δ/σ)_max_ < 0.001
106 parameters	Δρ_max_ = 0.38 e Å^−^^3^
0 restraints	Δρ_min_ = −0.56 e Å^−^^3^

### Special details

**Table:**

Geometry. All e.s.d.'s (except the e.s.d. in the dihedral angle between two l.s. planes) are estimated using the full covariance matrix. The cell e.s.d.'s are taken into account individually in the estimation of e.s.d.'s in distances, angles and torsion angles; correlations between e.s.d.'s in cell parameters are only used when they are defined by crystal symmetry. An approximate (isotropic) treatment of cell e.s.d.'s is used for estimating e.s.d.'s involving l.s. planes.
Refinement. Refinement of *F*^2^ against ALL reflections. The weighted *R*-factor *wR* and goodness of fit *S* are based on *F*^2^, conventional *R*-factors *R* are based on *F*, with *F* set to zero for negative *F*^2^. The threshold expression of *F*^2^ > σ(*F*^2^) is used only for calculating *R*-factors(gt) *etc*. and is not relevant to the choice of reflections for refinement. *R*-factors based on *F*^2^ are statistically about twice as large as those based on *F*, and *R*- factors based on ALL data will be even larger.

### Fractional atomic coordinates and isotropic or equivalent isotropic displacement parameters (Å^2^)

**Table:**

	*x*	*y*	*z*	*U*_iso_*/*U*_eq_	
Zn1	0.0000	−0.23661 (3)	−0.2500	0.01455 (15)	
Ca1	0.5000	−0.51637 (6)	0.2500	0.01865 (18)	
O1	0.2538 (3)	−0.34567 (15)	−0.11362 (14)	0.0200 (3)	
O2	0.5157 (3)	−0.35902 (17)	0.09520 (15)	0.0244 (4)	
O3	0.7153 (3)	−0.29814 (17)	−0.11799 (15)	0.0221 (3)	
O4	0.8547 (3)	−0.10913 (16)	−0.16958 (16)	0.0209 (3)	
O5	0.0955 (3)	−0.4370 (2)	0.14219 (19)	0.0345 (4)	
H5A	0.0534	−0.3562	0.1510	0.041*	
H5B	−0.0270	−0.4882	0.1110	0.041*	
C1	0.4111 (4)	−0.2941 (2)	−0.00731 (19)	0.0144 (4)	
C2	0.7341 (3)	−0.1716 (2)	−0.11904 (19)	0.0156 (4)	
C3	0.4642 (3)	−0.1423 (2)	−0.00489 (18)	0.0138 (4)	
C4	0.6098 (3)	−0.0840 (2)	−0.05841 (18)	0.0139 (4)	
C5	0.3573 (4)	−0.0571 (2)	0.05340 (18)	0.0160 (4)	
H5	0.2618	−0.0953	0.0898	0.019*	
O6	1.0000	0.1852 (4)	−0.2500	0.0787 (13)	
H6	0.9216	0.1282	−0.2269	0.094*	

### Atomic displacement parameters (Å^2^)

**Table:**

	*U*^11^	*U*^22^	*U*^33^	*U*^12^	*U*^13^	*U*^23^
Zn1	0.0131 (2)	0.0155 (2)	0.0160 (2)	0.000	0.00726 (16)	0.000
Ca1	0.0200 (3)	0.0125 (3)	0.0166 (3)	0.000	0.0012 (2)	0.000
O1	0.0177 (7)	0.0131 (7)	0.0220 (7)	−0.0011 (5)	0.0016 (6)	0.0009 (5)
O2	0.0274 (8)	0.0226 (8)	0.0185 (7)	0.0040 (6)	0.0054 (6)	0.0062 (6)
O3	0.0241 (8)	0.0164 (7)	0.0272 (8)	−0.0003 (7)	0.0123 (7)	−0.0057 (6)
O4	0.0220 (8)	0.0206 (7)	0.0271 (8)	−0.0008 (6)	0.0171 (7)	−0.0036 (6)
O5	0.0247 (8)	0.0326 (9)	0.0434 (11)	0.0056 (8)	0.0118 (8)	0.0062 (8)
C1	0.0128 (9)	0.0134 (9)	0.0171 (9)	0.0011 (7)	0.0063 (7)	−0.0012 (7)
C2	0.0110 (8)	0.0198 (10)	0.0144 (9)	0.0009 (8)	0.0040 (7)	−0.0045 (7)
C3	0.0118 (8)	0.0148 (9)	0.0136 (9)	−0.0016 (7)	0.0043 (7)	−0.0032 (7)
C4	0.0121 (8)	0.0153 (10)	0.0136 (9)	−0.0013 (7)	0.0050 (7)	−0.0031 (7)
C5	0.0156 (9)	0.0174 (10)	0.0160 (9)	−0.0031 (8)	0.0077 (7)	−0.0023 (7)
O6	0.071 (3)	0.040 (2)	0.085 (3)	0.000	−0.005 (2)	0.000

### Geometric parameters (Å, º)

**Table:**

Zn1—O4^i^	1.9701 (15)	O3—C2	1.243 (3)
Zn1—O4^ii^	1.9701 (15)	O3—Ca1^v^	2.3618 (17)
Zn1—O1^iii^	1.9754 (15)	O4—C2	1.273 (3)
Zn1—O1	1.9754 (15)	O4—Zn1^vii^	1.9701 (15)
Ca1—O2^iv^	2.3571 (17)	O5—H5A	0.8500
Ca1—O2	2.3571 (17)	O5—H5B	0.8500
Ca1—O3^v^	2.3618 (17)	C1—C3	1.517 (3)
Ca1—O3^vi^	2.3618 (17)	C2—C4	1.497 (3)
Ca1—O5^iv^	2.4003 (19)	C3—C5	1.391 (3)
Ca1—O5	2.4003 (19)	C3—C4	1.403 (3)
Ca1—O1^v^	2.9172 (16)	C4—C5^viii^	1.392 (3)
Ca1—O1^vi^	2.9172 (16)	C5—C4^viii^	1.392 (3)
O1—C1	1.280 (2)	C5—H5	0.9300
O1—Ca1^v^	2.9172 (16)	O6—H6	0.8500
O2—C1	1.231 (3)		
			
O4^i^—Zn1—O4^ii^	101.57 (9)	O3^v^—Ca1—O1^vi^	72.21 (5)
O4^i^—Zn1—O1^iii^	109.22 (7)	O3^vi^—Ca1—O1^vi^	66.20 (5)
O4^ii^—Zn1—O1^iii^	110.65 (7)	O5^iv^—Ca1—O1^vi^	123.51 (6)
O4^i^—Zn1—O1	110.65 (7)	O5—Ca1—O1^vi^	75.32 (6)
O4^ii^—Zn1—O1	109.22 (7)	O1^v^—Ca1—O1^vi^	124.96 (6)
O1^iii^—Zn1—O1	114.72 (9)	C1—O1—Zn1	123.33 (14)
O2^iv^—Ca1—O2	98.59 (9)	C1—O1—Ca1^v^	108.26 (12)
O2^iv^—Ca1—O3^v^	140.78 (6)	Zn1—O1—Ca1^v^	105.21 (6)
O2—Ca1—O3^v^	103.07 (6)	C1—O2—Ca1	149.04 (15)
O2^iv^—Ca1—O3^vi^	103.07 (6)	C2—O3—Ca1^v^	141.97 (13)
O2—Ca1—O3^vi^	140.78 (6)	C2—O4—Zn1^vii^	111.98 (13)
O3^v^—Ca1—O3^vi^	79.77 (9)	Ca1—O5—H5A	123.2
O2^iv^—Ca1—O5^iv^	77.30 (6)	Ca1—O5—H5B	125.0
O2—Ca1—O5^iv^	78.37 (7)	H5A—O5—H5B	109.3
O3^v^—Ca1—O5^iv^	138.88 (7)	O2—C1—O1	124.0 (2)
O3^vi^—Ca1—O5^iv^	75.09 (7)	O2—C1—C3	117.63 (18)
O2^iv^—Ca1—O5	78.37 (7)	O1—C1—C3	118.31 (17)
O2—Ca1—O5	77.30 (6)	O3—C2—O4	123.82 (17)
O3^v^—Ca1—O5	75.09 (7)	O3—C2—C4	119.71 (17)
O3^vi^—Ca1—O5	138.88 (7)	O4—C2—C4	116.47 (18)
O5^iv^—Ca1—O5	142.29 (10)	C5—C3—C4	118.95 (18)
O2^iv^—Ca1—O1^v^	152.50 (5)	C5—C3—C1	116.74 (18)
O2—Ca1—O1^v^	73.48 (5)	C4—C3—C1	124.31 (17)
O3^v^—Ca1—O1^v^	66.20 (5)	C5^viii^—C4—C3	119.66 (18)
O3^vi^—Ca1—O1^v^	72.21 (5)	C5^viii^—C4—C2	119.30 (18)
O5^iv^—Ca1—O1^v^	75.32 (6)	C3—C4—C2	121.03 (18)
O5—Ca1—O1^v^	123.51 (6)	C3—C5—C4^viii^	121.39 (18)
O2^iv^—Ca1—O1^vi^	73.48 (5)	C3—C5—H5	119.3
O2—Ca1—O1^vi^	152.50 (5)	C4^viii^—C5—H5	119.3
			
O4^i^—Zn1—O1—C1	−44.54 (17)	Ca1^v^—O3—C2—O4	−82.2 (3)
O4^ii^—Zn1—O1—C1	66.46 (16)	Ca1^v^—O3—C2—C4	97.8 (2)
O1^iii^—Zn1—O1—C1	−168.66 (17)	Zn1^vii^—O4—C2—O3	1.6 (2)
O4^i^—Zn1—O1—Ca1^v^	−169.11 (6)	Zn1^vii^—O4—C2—C4	−178.32 (13)
O4^ii^—Zn1—O1—Ca1^v^	−58.11 (7)	O2—C1—C3—C5	−77.1 (2)
O1^iii^—Zn1—O1—Ca1^v^	66.77 (4)	O1—C1—C3—C5	100.1 (2)
O2^iv^—Ca1—O2—C1	−96.5 (3)	O2—C1—C3—C4	103.6 (2)
O3^v^—Ca1—O2—C1	50.6 (3)	O1—C1—C3—C4	−79.2 (2)
O3^vi^—Ca1—O2—C1	140.4 (3)	C5—C3—C4—C5^viii^	−0.6 (3)
O5^iv^—Ca1—O2—C1	−171.5 (3)	C1—C3—C4—C5^viii^	178.61 (17)
O5—Ca1—O2—C1	−20.5 (3)	C5—C3—C4—C2	178.36 (17)
O1^v^—Ca1—O2—C1	110.5 (3)	C1—C3—C4—C2	−2.4 (3)
O1^vi^—Ca1—O2—C1	−25.9 (4)	O3—C2—C4—C5^viii^	176.38 (17)
Ca1—O2—C1—O1	−45.2 (4)	O4—C2—C4—C5^viii^	−3.7 (3)
Ca1—O2—C1—C3	131.8 (2)	O3—C2—C4—C3	−2.6 (3)
Zn1—O1—C1—O2	154.42 (16)	O4—C2—C4—C3	177.33 (17)
Ca1^v^—O1—C1—O2	−82.4 (2)	C4—C3—C5—C4^viii^	0.6 (3)
Zn1—O1—C1—C3	−22.6 (2)	C1—C3—C5—C4^viii^	−178.66 (17)
Ca1^v^—O1—C1—C3	100.63 (17)		

Symmetry codes: (i) *x*−1, *y*, *z*; (ii) −*x*+1, *y*, −*z*−1/2; (iii) −*x*, *y*, −*z*−1/2; (iv) −*x*+1, *y*, −*z*+1/2; (v) −*x*+1, −*y*−1, −*z*; (vi) *x*, −*y*−1, *z*+1/2; (vii) *x*+1, *y*, *z*; (viii) −*x*+1, −*y*, −*z*.

### Hydrogen-bond geometry (Å, º)

**Table:**

*D*—H···*A*	*D*—H	H···*A*	*D*···*A*	*D*—H···*A*
O6—H6···O4	0.85	2.49	3.255 (4)	151
O5—H5*A*···O6^viii^	0.85	2.11	2.914 (4)	157
O5—H5*B*···O1^ix^	0.85	2.16	2.951 (2)	156

Symmetry codes: (viii) −*x*+1, −*y*, −*z*; (ix) −*x*, −*y*−1, −*z*.
